# Differential analysis of quantitative proteome and acetyl-proteome profiling between premenopausal and postmenopausal ovarian tissues

**DOI:** 10.1186/s12014-018-9214-0

**Published:** 2018-11-16

**Authors:** Jinling Yi, Huatianshu Hu, Peipei Shi, Song Shi, Junda Zhao, Linna Xu, Weining Yang, Bin Li, Jin Zhu, Shien Zou

**Affiliations:** 1 Department of Gynecology, The Fifth Affiliated Hospital of Xin Jiang Medical University, Number 118, Henan Road, Ürümqi, Xinjiang Uygur Autonomous Region China; 2 Department of Gynecology, Obstetrics and Gynecology Hospital, Fudan University, Number 128, Shenyang Road, Shanghai, 200011 China; 3 Department of Function Inspection, The Fifth Affiliated Hospital of Xin Jiang Medical University, Number 118, Henan Road, Ürümqi, Xinjiang Uygur Autonomous Region China; 4 Department of Gynecology, The First Affiliated Hospital of Xinjiang Medical University, Number 137, South Liyushan Road, Ürümqi, Xinjiang Uygur Autonomous Region China

**Keywords:** Human ovarian, Menopause, Proteome, Acetyl-proteome

## Abstract

**Background:**

Natural menopause is always accompanied by specific signs and symptoms, suggesting physiological changes in this peoriod. However, no systematic study has assessed the changes at molecular level in the ovaries during the menopausal transition so far. This study integrated quantitative proteome and acetyl-proteome to comprehensively uncover the changes of ovarian protein and protein-acetylation profiles in this transitional period. The findings would provide novel insights into the biology of menopause and help relieve and treat the associated signs and symptoms, further improving the women’s health care.

**Methods:**

Freshly thawed ovarian tissue samples obtained from premenopausal and postmenopausal women were assessed with Tandem Mass Tags for the quantitative analysis of the global profile and acetyl-proteomes by 2-dimensional separation and LC–MS/MS.

**Results:**

Comprehensively, 4210 types of protein, with 3551 types quantifiable were detected. 3047 acetylated sites in 1583 types of protein with 2256 quantifiable in 1248 proteins were detected. By comparing the global and acetylated proteome profiles for postmenopausal women and premenopausal women, 151 types of proteins were found upregulated and 65 were downregulated, along with 23 acetylated sites upregulated and 220 sites downregulated. For Immune response, the complement and coagulation cascades plus the citrate cycle and cellular detoxification were found to be significantly enhanced, while the extracellular structure and matrix organization, ECM-receptor interactions plus the infections were markedly suppressed. In addition, the amino acids around the acetylated sites were enriched by motif analysis, which can help us uncover amino acid sequence and search for the specific target in the subsequent study.

**Conclusion:**

Global and acetylated proteome Profiles in ovary differ between the premenopausal and postmenopausal groups. These proteomic-level changes may offer some potential biological markers to identify the pathological changes in ovary and help relieve and treat the associated signs and symptoms, and ultimately improve women’s health care.

**Electronic supplementary material:**

The online version of this article (10.1186/s12014-018-9214-0) contains supplementary material, which is available to authorized users.

## Background

Menopause is defined as the permanent termination of menstruation due to exhaustion of the resting primordial follicle pool and loss of ovarian follicular activity, and usually occurs at 51 years of age averagely (ranging from 40 to 60 years) [1]. Despite it’s a natural process, menopause significantly affects women, both physically and emotionally, which is closely associated with psychosocial issues of midlife [2]. The menopausal manifestations most reported are hot flushes and night sweats. In addition, menopause may also cause bone loss, breast diseases, problems in cardiovascular system, mood and cognitive dysfunctions, sexual issues, etc. [3, 4]. The risk of ovarian cancer is also elevated during perimenopause [5]. Epidemiological surveys have evaluated the side effects of menopause [6], while biological transformations during menopausal transition are rarely assessed [7], so there is a lack of systematic understanding of menopause.

Protein modifications including methylation, phosphorylation, acetylation,ubiquitination, etc., are closely related to the pathogenesis and development of diseases,. For all kinds of protein modifications, Protein acetylation is particularly important as reported, [8] so we hypothesized that menopause might be associated with acetyl-proteome. Quantitative proteomics, a powerful approach in analyzing global proteomic dynamics, can identify thousands of proteins at the tissue, cellular, and subcellular levels [9, 10]. Combined with specific enrichment methods for modified peptides, such as phosphorylation [11], ubiquitination, and acetylation [12], it can also help assess post-translational modifications of the proteome with a high resolution [13]. Beyond qualitative identification of thousands of proteins in a specific sample, it is equally crucial to quantitate them for the differences of protein expression [14, 15], which is critical in exploring and understanding protein kinetics and biological functions at the molecular level [16].

Isobaric mass tags are often applied for relative quantification with multiplex capabilities and high throughput [17, 18]. Tandem mass tag (TMT) is commercially available to simultaneously analyze 4, 6 or 8 samples [19, 20]. TMT is composed of a mass reporter, a mass normalizer, and a reactive moiety [19, 21]. By HCD-induced tag cleavage, the tag is used to quantitate relative peptide intensities in the first dimensional MS, and peptide fragment ions are sequenced to identify of proteins in the second dimensional MS [22].

This study applied TMT-labeled LC–MS/MS, combined with antibody-affinity purification for acetylated peptides, to profile the global proteome and acetyl-proteome in premenopausal and postmenopausal ovarian tissues. By comparing protein expression in postmenopausal and premenopausal women, we systematically analyzed changes at molecular level from premenopause to postmenopause, and calculated the regularity of sequence features around the acetylation sites. The proteins and acetylated sites significantly different between pre and post-menopausal women could be potential targets for the development of therapeutics to relieve and treat perimenopausal symptoms. Our current study aimed to systematically assess the changes at molecular level in the ovaries during perimenopausal transition, promoting the biological understanding of this natural process and finally benefits women’s health.

## Methods

### Collection of human ovarian tissues

Patients meet inclusion criteria as below were considered eligible for sample collection. (1) age from 45 to 60 year-old; (2) No steroid hormone drugs taken for at least 6 months preceding the surgery; (3) premenopausal women with benign ovarian cysts with cystectomy or adnexectomy done or postmenopausal women with adnexectomy done for benign disease. Normal ovarian cortical tissue samples were collected carefully without affecting the pathological diagnosis. All subjects had no morbid diseases such as cancer or organ failures. In addition, every subject agreed to participate in this study with signed informed consent. Exclusion criteria included patients with cancer, patients with mental disabilities, and patients with potential risk during blood drawing; those who quit in the middle were also excluded, as well as those who were not compliant with poor follow-up.

Based on the criteria above, fresh-frozen ovarian samples, which were collected from three women with regular menstruation aged at 46, 48 and 51, respectively, and another three women aged at 52, 59 and 59 menopaused for 2, 6, and 8 years, respectively, were sent for both global and acetylated proteomic analyses. The tissue specimens were kept at − 80 °C before use, and pre-processed by pathologists from Obstetrics and Gynecology Hospital of Fudan University. This study was approved by ethics committee of Obstetrics and Gynecology Hospital of Fudan University, China.

### Reagents

Dithiothreitol, ethylenediamine tetraacetic acid (EDTA), iodoacetamide, nicotinamide (NAM), quagolomycin A (TSA), tetraethylammonium bromide (TEAB) and urea were purchased from Sigma (Santa Clara, CA). Acetonitrile, C18 columns (Waters, Milford, Massachusetts, USA), distilled water (Fisher Chemical, Waltham, Massachusetts, USA), formic acid (Fluka, Santa Clara, CA, USA), protease inhibitor (Calbiochem, Darmstadt, Germany), trifluoroacetic acid (Sigma-Aldrich, Santa Clara, CA, USA), and trypsin (Promega, Madison, Wisconsin, USA) were utilized in the experiments. The BCA assay kit was supplied by Beyotime biotechnology (Shanghai, China). The TMT kit and mass calibration standards were produced by Thermo Fisher Scientific (Waltham, Massachusetts, USA). Additional chemicals were purchased in China.

### Protein extraction and trypsin digestion

The samples were homogenized in liquid nitrogen and transferred into tubes. Then, four portions of lysis buffer (8M urea, 1% Protease Inhibitor Cocktail) were added to the sample, which was stood for 30 min. In PTM experiments, inhibitors were also added to the lysis buffer, e.g. 3 μM TSA and 50 mM NAM for acetylation. After sonication thrice on ice on a high intensity ultrasonic processor (Scientz), the sample was fully lysed on ice for another 30 min. The remaining cell debris were precipitated and removed by centrifugation at 12,000*g* at 4 °C for 10 min. The resulting supernatant was obtained for protein level test using the BCA kit.

The proteins were then digested with 5 mM dithiothreitol for 30 min at 56 °C and submitted to alkylation using 11 mM iodoacetamide for 15 min at room temperature away from light. Then, 100 mM TEAB was added to urea to dilute till < 2 M in the protein solution. Next, trypsin dissolved in buffer was added for overnight digestion at the trypsin to protein mass ratio of 1:50; a second digestion was carried out for 4 h at trypsin to protein mass ratio at 1:100 [23].

### TMT labeling

The digested peptides were submitted to desalting using a Strata X C18 SPE column (Phenomenex), as directed by the manufacturer. Briefly, the peptide mixture in 0.5M TEAB and TMT reagent were further treated with acetonitrile, submitted to incubation for 2 h at room temperature, desalting, and drying [24, 25].

### Peptide separation by high performance liquid chromatography (HPLC)

The tryptic peptides were separated by high pH reverse-phase HPLC with a C18 column (5 μm particle size, 10 mm ID, 250 mm length). For elution, a gradient of 8–32% acetonitrile (pH 9.0) was employed over 60 min into 60 fractions. To generate eighteen total fraction of proteome is combined (final fraction 1 = 1, 19, 37, 55, 73; final fraction 2 = 2, 20, 38, 56, 74; final fraction 3 = 3, 21, 39, 57, 75; ………; final fraction 18 = 18, 36, 54, 72). To generate six total fraction of acetylation is combined (final fraction 1 = 1, 7, 13, 19, 25, 31, 37, 43, 49, 55, 61, 67, 73, 79; final fraction 2 = 2, 8, 14, 20, 26, 32, 38, 44, 50, 56, 62, 68, 74, 80;………; final fraction 6 = 6, 12, 18, 24, 30, 36, 42, 48, 54, 60, 66, 72, 78). After sample combination into 18 and 6 fractions, respectively, the specimens were submitted to drying by vacuum centrifugation.

### Affinity enrichment of acetylated peptides

For the enrichment of acetyl-modified peptides, tryptic peptides in NETN buffer (100 mM NaCl, 1 mM EDTA, 50 mM Tris–HCl, 0.5% NP-40, pH 8.0) were submitted to incubation in presence of acetyl-antibody beads (Lot number PTM104, PTM Bio) at 4 °C overnight. Bound peptides were dissociated by treatment with 0.1% trifluoroacetic. After vacuum drying, the peptide specimens were desalted with C18 ZipTips (Millipore) as directed by the manufacturer, for LC–MS/MS [26, 27].

### LC–MS/MS

Tryptic peptide specimens were formulated in 0.1% formic acid (solvent A), and loaded onto a home-made reversed-phase analytical column (15 cm length, 75 μm ID). The gradient was increased from 6 to 23% solvent B (0.1% formic acid in 98% acetonitrile) over 26 min, 23–35% in 8 min, and increased in 3 min to 80%, maintained for another 3 min (flow rate, 400 nL/min) on an EASY-nLC 1000 UPLC system.

NSI source and tandem mass spectrometry (MS/MS) on Q Exactive™ Plus (Thermo) coupled to UPLC were used for analysis (electrospray voltage, 2.0 kV; m/z range, 350–1800). Non altered peptides were detected in the Orbitrap at a resolution of 70,000. Peptide selection for MS/MS was carried out with NCE setting as 28, with fragment detection in the Orbitrap (resolution, 17,500). A data-dependent procedure alternating between 1 MS scan and 20 MS/MS scans was adopted, with 15.0 s dynamic exclusion. Automatic gain control (AGC) was 5E4, with a fixed first mass of 100 m/z [28].

### Data analysis

Raw MS/MS data processing was performed with the Maxquant search engine (v.1.5.2.8) [29]; spectrum analysis was performed with the SwissProt (human, 20,130 sequences) and reverse decoy databases. At most 4 missing cleavages were allowed. The least length of peptides was 7 amino acids; the most modifications were 5. In the initial and main searches, mass tolerance values were 20 ppm and 5 ppm, respectively, and 0.02 Da fragment was tolerated. Alkylation on cysteine was considered a stable modification, while N-linked and lysine acetylation as well as oxidation on Met were considered variable modifications. FDR was adjusted to < 1% for peptide and protein [30]. Minimum score for the modified peptides of > 40. The quantification method was based on TMT-6plex.

Gene Ontology (GO) (http://www.ebi.ac.uk/GOA/) was used as recently described for functional annotation [31]. For proteins not annotated by UniProt-GOA, InterProScan was employed to annotate protein’s GO function considering sequence alignment. The proteins were assigned to 3 GO categories, including biological process, cellular compartment, and molecular function. In all categories, a two-tailed Fisher’s exact test was employed to assess enrichment of differentially expressed proteins. Corrected *P* < 0.05 indicated statistical significance.

The functional domains of the obtained proteins were annotated by InterProScan on the basis of sequence alignment, using the InterPro domain database (http://www.ebi.ac.uk/interpro/) [32]. Corrected *P* < 0.05 indicated statistical significance. Here, subcellular localization was predicted by Wolfpsort.

Kyoto Encyclopedia of Genes and Genomes (KEGG) was employed for protein pathway annotation [33]. A two-tailed Fisher’s exact test was applied for assessing enriched differentially expressed proteins. Corrected *P* < 0.05 indicated statistical significance. Hierarchical categories were obtained based on the KEGG database.

Further hierarchical clustering according to protein functions was performed as described previously [34]. Briefly, all enriched categories were filtered to have at least one cluster with *P* < 0.05. The resulting P value matrix was transformed by x = − log_10_(P), and z-transformed for various functional categories. The z scores were clustered by one-way hierarchical clustering (Euclidean distance, average linkage clustering) in Genesis. A heat map was generated with the “heatmap.2” function in “gplots” (R-package) for visualizing cluster members.

## Results and discussion

### Identification and quantitation of global- and acetyl-proteomes in ovary

**Fig. 1:**
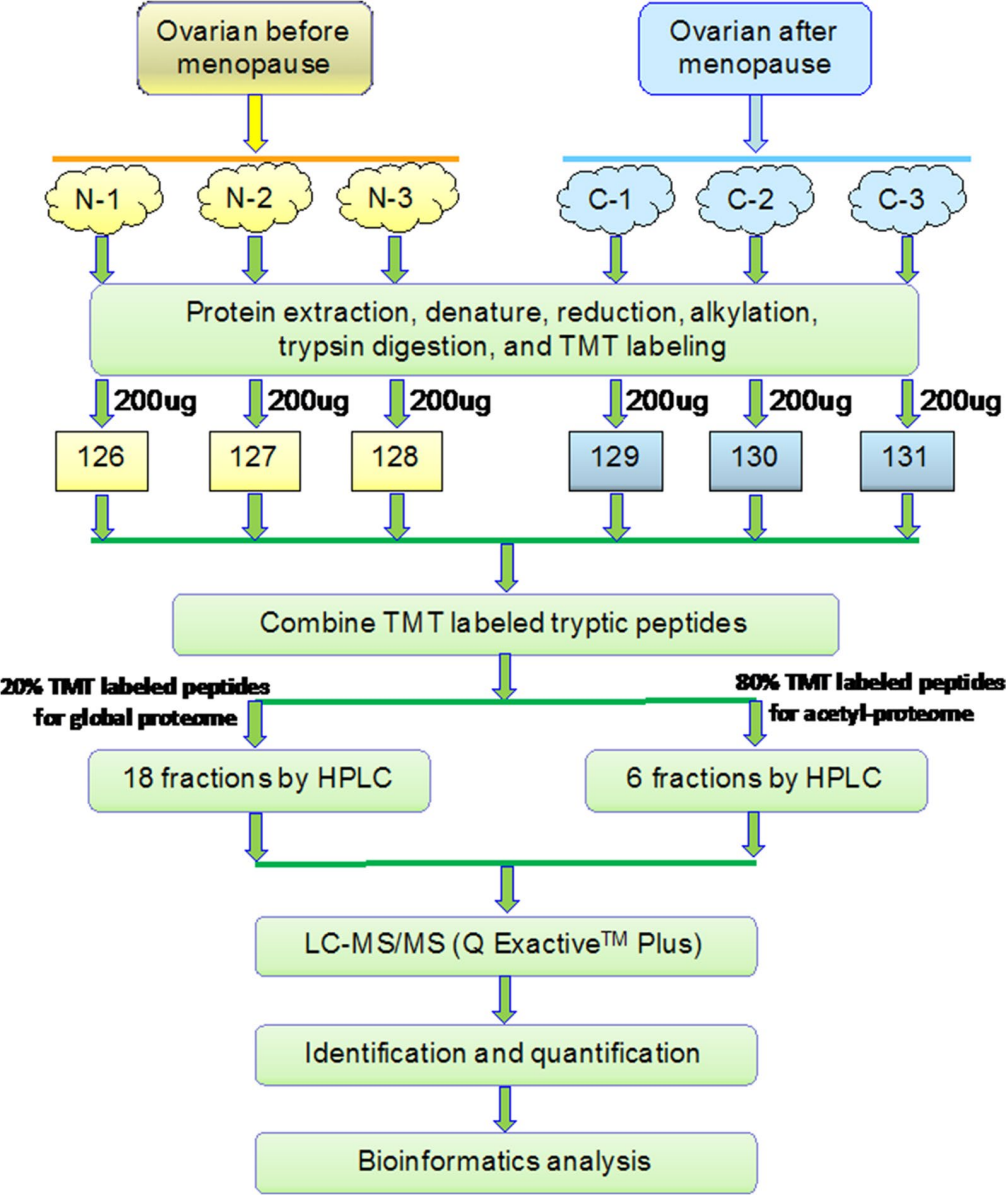
Quantification strategy for global and acetylated proteomes in the ovary during premenopause and postmenopause

We simultaneously identified and quantified the global and acetyl-proteome profiling by TMT labeling in three replicates using 3 ovarian premenopausal samples and 3 postmenopausal ovarian samples, respectively. The overall strategy is illustrated in Fig. 1. Notably, the quantitation of global and acetyl-proteins according to TMT-labeling and LC–MS/MS usually comprised of the following steps. First, recovery and TMT labeling of the 6groups of digested peptides from ovarian proteins; second, combine the 6 groups of labeled peptides equally; thirdly, for global proteome analysis, separation to 60 fractions by HPLC and pooling to 18 fractions were done, with direct analysis by high-resolution LC–MS/MS after desalting; for acetyl-proteome analysis, separation to 60 fractions by HPLC, pooling to 6 fractions, enrichment of acetylated peptides by antibody-based modification enrichment, and desalting and analysis by mass spectrometry were done. By applying this approach, expression changes of global and acetylated proteins could be assessed simultaneously.

TMT-labeled LC–MS/MS combined with pre-fractionation by HPLC allows for comprehensive proteome profiling with deep coverage. Combined with the acetylated peptide enrichment method, this strategy helped assess the degree of global proteome changes, as well as acetyl-protein expression changes, simultaneously.

For the global proteome in the ovary, 4210 proteins were identified, including 3551 with quantification in triplicate (Additional file 1: Table S1). The number of differentially expressed proteins reached 5–10% of the quantifiable proteins in total [35, 36]. Comparing the protein expression levels in postmenopausal and premenopausal groups at the threshold of 1.3 fold-change and *t* test *P* < 0.05, 151 proteins were found up-regulated and 65 were down-regulated in all (Additional file 2: Table S2).

For acetyl-proteome analysis after acetylated peptide enrichment, 3047 acetylated sites in 1583 proteins were uncovered, including 2256 acetylated sites in 1248 proteins with quantification in triplicate (Additional file 3: Table S3). Similarly, based on a threshold of 1.3 fold change and *t* test *P* < 0.05 as standards, 23 up-regulated acetylated sites in 19 proteins, and 220 down-regulated acetylated sites in 186 proteins comparing postmenopause with premenopause were found (Additional file 4: Table S4), indicating that there were multiple acetylated sites for some proteins. The 12 acetylated proteins most significantly different in ovarian tissues in the two groups are listed in Table 1. Standard deviation of the analysis was displayed in Additional file 1.

**Table 1:** The top 12 differentially expressed acetylated proteins in the ovary after postmenopause compared to pre-menopause

Protein description	Modified sequence	Postmeno/permeno ratio	Postmeno/permeno *P* value
Neuroblast differentiation-associated protein AHNAK	VTFPK(1)MK	2.37	4.7e−6
Polymerase I and transcript release factor	K(1)SFTPDHVVYAR	2.16	2.7e−5
Dihydrolipoyllysine-residue succinyltransferase component of 2-oxoglutarate dehydrogenase complex	HK(1)EAFLK(1)K	2.15	1.3e−4
Thy-1 membrane glycoprotein	K(1)HVLFGTVGVPEHTYR	1.81	4.2e−4
WD repeat-containing protein mio	EFVPK(1)HAR	1.70	6.9e−4
Complement C5	YK(1)HSVVK	0.61	2.1e−4
Carbonic anhydrase 1	VGEANPK(1)LQK	0.59	1.6e−4
Ig gamma-4 chain C region	PSNTK(1)VDK	0.57	6.9e−5
Glutathione S-transferase Mu4	K(1)HNLCGETEEEKIR	0.50	1.7e−4
Transferrin receptor protein 1	GVEPK(1)TECER	0.43	1.8e−4
Interferon-induced protein with tetratricopeptide repeats 1	ALELLK(1)K	0.32	2.9e−2
Interferon-induced protein with tetratricopeptide repeats 1	YAAK(1)FYR	0.27	4.5e−2

To assess repeatability among the 3 biological replicates, scatter plots were generated between two samples, separately, and repeatability ranged from 0.55 to 0.61 for the global proteome (Additional file 5: Fig. S1), and from about 0.50 to 0.54 for the acetyl-proteome, slightly reduced compared with the global proteome (Additional file 6: Fig. S2). This indicated that, considering the difference among clinical samples, we achieved satisfactory repeatability among the three independent experiments.

To validate the specificity of the antibody, some extra acylation sites were searched with the MS/MS data in our study. Compared with 3047 acetylation sites, only 35 succinylation sites and 43 malonylation sites were detected in the MS/MS raw data  (Additional file 2: Table S2). So we concluded that the antibodies used in this study is highly specific for succinylation. Additionally, more than 79% (2957) of 3713 peptides identified were found to be acetylated, which may suggest the efficiency of our modified peptides enrichment. Reproducibility of acetylation analysis was shown in Additional file 3: Table S3.

### Functional analysis of differential proteins and acetylated proteins between perimenopausal and postmenopausal women

To explore the functional annotation of the differentially expressed proteins, their biological processes, cellular components and molecular functions were compared between postmenopausal and premenopausal specimens based on Gene Ontology. Generally, the most significantly different biological processes included cellular, single-organism, and metabolic processes, as well as biological regulation, which are generally the building blocks of life. The cellular components mainly focuse on cell, organelle, and extracellular compartments. The most significant molecular functions included protein binding and catalytic activity. The findings above also applied to differential acetyl-proteins detected. Further, we performed enrichment analysis beyond the main three categories for the retrieved differential proteins and acetyl-proteins.

**Fig. 2:**
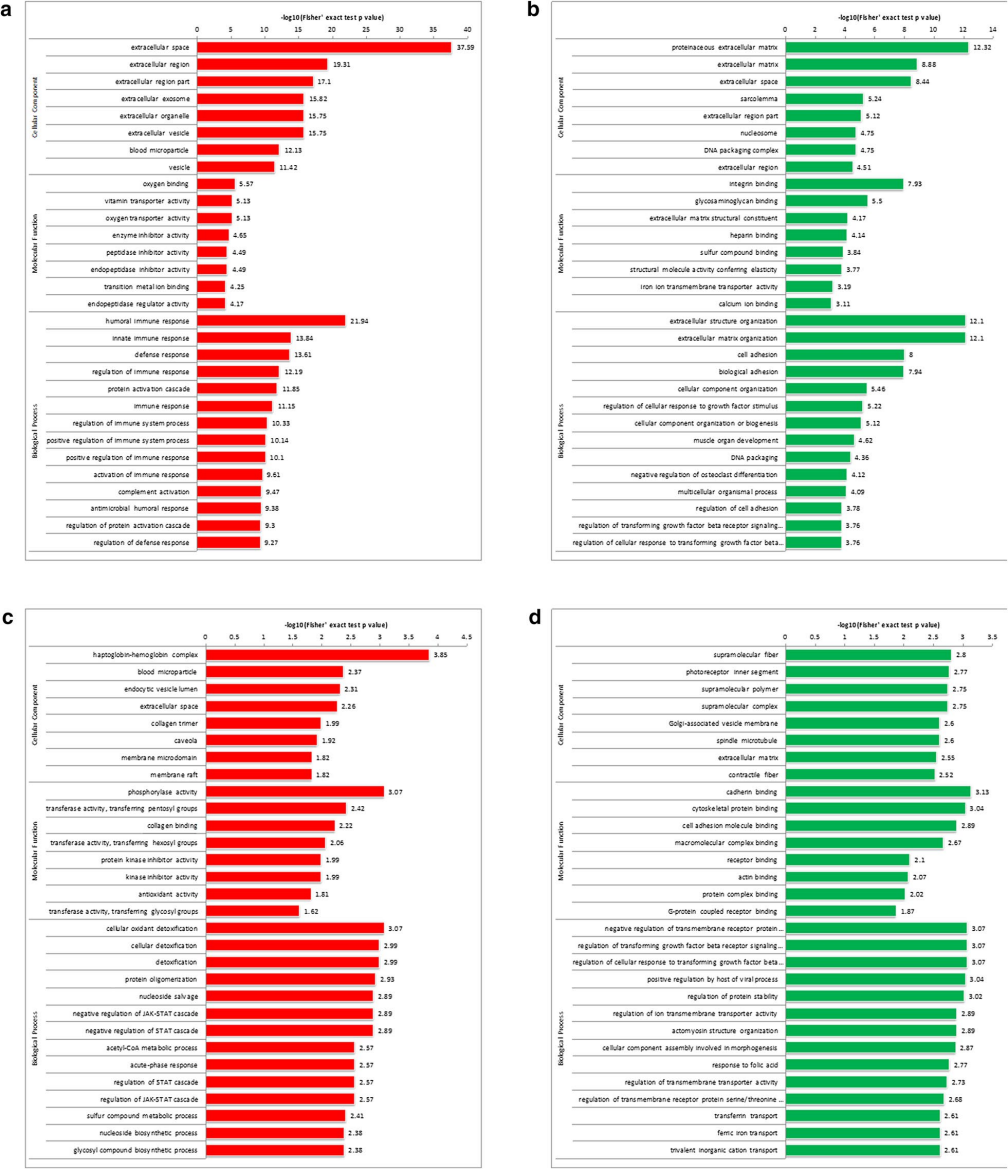
Functional enrichment of differential proteins by Gene Ontology. Gene Ontology enrichment of upregulated proteins (**a**), downregulated proteins (**b**), upregulated acetylated proteins (**c**), and downregulated acetylated proteins (**d**). Values of the horizontal axis are the negative log transformed P values (P < 0.05)

Surprisingly, there was a significant difference between up- and down-regulated proteins, for both global and acetylated proteins. Compared to ovarian premenopausal samples, the most significantly enriched cellular component was extracellular space for up-regulated proteins from postmenopause (Fig. 2a), while down-regulated proteins were generally found in the extracellular matrix (Fig. 2b). The molecular functions mainly found were for increased oxygen binding or decreased integrin binding (Fig. 2a, b). Humoral immune response was significantly increased, as well as immune response regulation, while extracellular structure and matrix organization was rapidly decreased, as well as cell adhesion (Fig. 2a, b). As for acetylated proteins, the most significantly increased functional modules were haptoglobin-hemoglobin complex, phosphorylase activity and cellular oxidant detoxification, accompanied with decreased supramolecular fiber, cadherin binding, and negative regulation of transmembrane receptor proteins (Fig. 2c, d). These findings suggested that at the global protein and protein modification levels, significant differences exist between the premenopausal and postmenopausal stages in ovary. It can be easily speculated that the ovary undergoes great functional adaptations to hormone deficiency and physiological changes.

### Pathway enrichment of differentially expressed and acetylated proteins between premenopausal and postmenopausal tissues

**Fig. 3:**
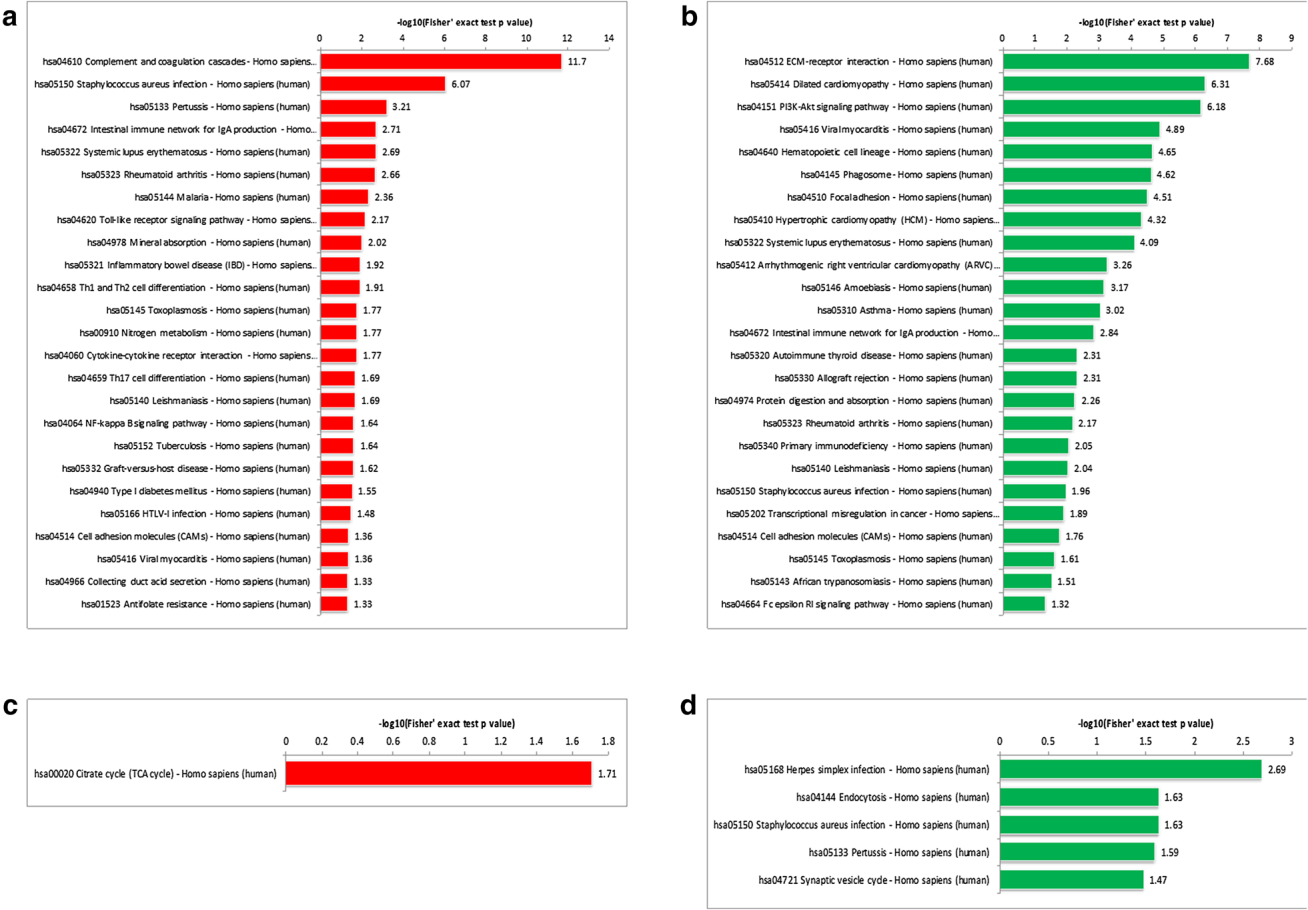
The enrichment pathways of differential proteins by the KEGG database. Pathway enrichment of upregulated proteins (**a**), downregulated proteins (**b**), upregulated acetylated proteins (**c**) and downregulated acetylated proteins (**d**). Values of the horizontal axis are the negative log transformed P values (P < 0.05)

Next, we explored pathway enrichment unveiled by differential proteins based on the KEGG database. 25 upregulated and 25 downregulated pathways selected from 151 upregulated and 65 downregulated proteins, for the pre and post-menopausal ovarian tissues. The most upregulated pathway in the ovary postmenopause was the complement and coagulation cascade, while the most downregulated was ECM-receptor interaction (Fig. 3a, b). For differential acetylated proteins, the only upregulated pathway was the citrate cycle, while the most downregulated were herpes simplex infection, endocytosis, and *Staphylococcus aureus* infection (Fig. 3c, d). These findings suggested that at postmenopause, coagulation is activated while energy supply is increased to meet the consumption demand in the ovary. With increased complement pathway, the ovary attempted to enhance immunity, while became more susceptible to infection, suggesting a process in which the ovary becomes increasingly weaker postmenopausally.

### Motif analysis for protein acetylation

To detect the amino acid sequences surrounding the acetylated sites, motif analysis was adopted to explore the amino acid sequences close to specific acetylated sites and statistics was done to analyze the results. The amino acids at the foreground and background of the acetylated sites were matched to whole size, and motif scores as well as fold changes were calculated. In this way, the amino acids near the acetylated sites were determined with their respective statistical significance as shown in Table 2, the most significant 15 AA sequences near the lysine acetylation site were obtained, including LKK, RKS, and KH.

**Table 2:** Feature sequences near the acetylated sites and statistics by motif analysis

Motif	Motif score	Foreground		Background		Fold increase
		Matches	Size	Matches	Size	
………LKK………	26.84	77	2237	5077	567,332	3.85
………RKS………	24.57	53	2910	2563	602,451	4.28
……….KH………	16	303	2540	14,778	582,110	4.7
……….KF………	16	317	2857	17,778	599,888	3.74
……….KN………	16	247	1358	24,766	449,103	3.3
……….KT………	16	260	1867	32,332	524,225	2.26
……….KR………	16	176	925	33,810	388,687	2.19
……….KS………	16	293	2160	38,030	562,255	2.01
……….KV………	16	186	1111	35,650	424,337	1.99
……….KK………	16	249	1607	42,790	491,893	1.78
…..K….K……….	12.34	115	749	26,765	354,877	2.04
…..R….K……….	12.03	86	634	19,331	328,112	2.3
……….KL………	9.17	127	463	45,844	283,454	1.7
……….KI………	7.88	85	548	25,327	308,781	1.89
……K…K……….	6.38	52	336	17,591	237,610	2.09

**Fig. 4:**
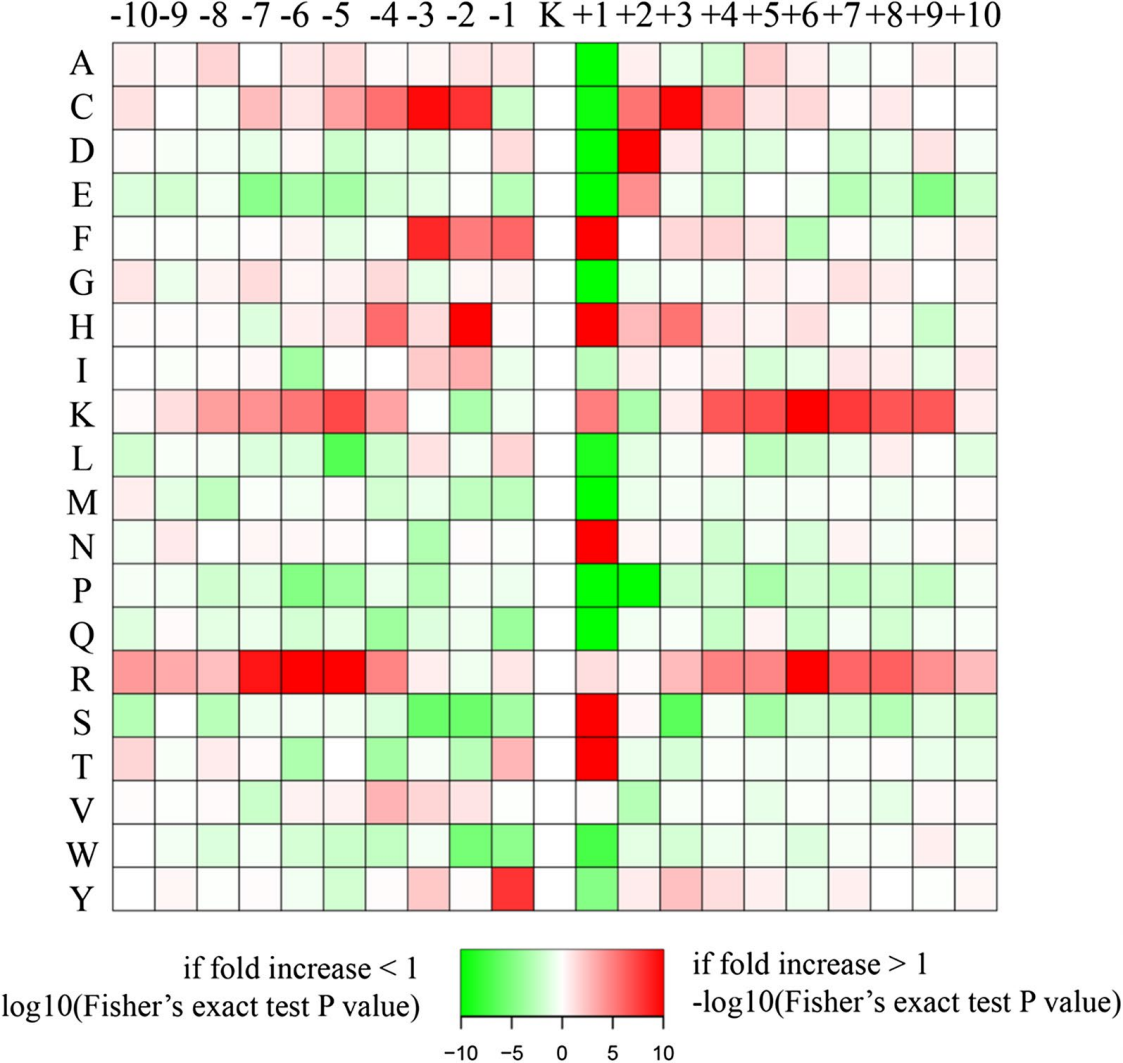
Heat map of motif enrichment for upstream and downstream amino acids of acetylated sites. Red, amino acid significantly enriched near the acetylated site; green, amino acid significantly declined near the acetylated site

To illustrate quantitative differences in sequence features close to the specific acetylated sites, we displayed the motif enrichment for upstream and downstream amino acids in the form of a heat map (Fig. 4). The first amino acid upstream the lysine acetylation site was most possibly tyrosine (Y), while the least possible was phenylalanine (F); the second amino acid upstream the lysine acetylation site was most possibly cysteine (C) or histidine (H); the third was cysteine (C) or phenylalanine (F). Similarly, the first amino acid downstream the lysine acetylation site was most possibly phenylalanine (F), histidine (H), asparagine (N), serine (S), or threonine(T); the second downstream was most possibly aspartic acid (D), cysteine (C), or glutamic acid (E); the third was cysteine (C) or histidine (H), etc.

### Significance of the current findings and future directions

Menopause is a natural process but accompanied with complex changes for women over 50 year-old; menopause, which is considered a turning point, may occur earlier in some women [37, 38]. According to the World Health Organization (WHO), an estimated one billion women will be over 60 year-old by the year 2050, suggesting that more than one billion women will be at the postmenopausal status, which is becoming a worldwide concern in public health [39]. Peri- and post-menopausal women often show multiple menopausal signs and symptoms, among which the most common during menopausal transition are hot flushes, night sweats, recurrent fatigue, and even memory loss and mild depression [40–42]. For severe symptoms, hormone therapy is used for alleviation or treatment [43, 44]. Currently, multiple surveys, meta-analyses and epidemiological studies have explored the potential factors related to menopause and its potential side effects on healthcare and life quality of elderly women and their families [45, 46]. However, the molecular mechanism behind menopause transition has been barely explored. Using clinical ovarian samples obtained from perimenopausal and postmenopausal women, we comprehensively identified and quantified the global and acetylated proteomes by large-scale LC–MS/MS with TMT labeling. As shown above, 4210 Proteins and 3047 acetylated sites in 1583 proteins were identified, as well as quantified 3551 proteins and 2256 acetylated sites in 1248 proteins, respectively There are some results reported about the total number of protein and acetylated peptide identifications are indeed high throughput and some are not, while it’s more important to find out differential expressed proteins. Besides, not all proteins can be quantified and there are many factors such as weak label signal intensity or interference [47]. This is the largest and most comprehensive proteome profiling in ovarian specimens from perimenopausal and postmenopausal women so far.

Based on high-resolution data, we analyzed the underlying biological significance. From the whole dataset, molecular functions, biological processes and cellular components were similar between premenopause and postmenopause. Surprisingly, there was significant differences when comparing differentially expressed proteins between the two groups, which constitutes the value of our findings. In detail, postmenopause, humoral immune responses and defense response against infections were rapidly increased because of hormone deficiency, while extracellular structure organization and cell adhesion were instead decreased, indicating cell functions are changed from building themselves to defending micro-environmental disturbances. In addition, the complement and coagulation cascade was significantly upregulated to protect the ovary from menopause. Similar changes were observed for the acetylated proteome. The haptoglobin-hemoglobin complex was apparently elevated, as well as the citrate cycle, but the ability to fight infection and endocytosis potential were sharply declined. These findings indicated great transformations of the ovary from premenopause to postmenopause, and some protein changes may be associated with menopause symptoms, and even constitute a sign of ovarian cancer.

We systematically analyzed the molecular dynamics in the ovary from premenopause to postmenopause, and the current findings may be a valuable reference for biological and clinical applications. However, some improvements can be made. First, considering individual differences, more clinical samples are needed to further confirm these findings; secondly, the differentially expressed and acetylated proteins should be further validated in more specific experiments such as targeted proteomics, Western blot, and ELISA; thirdly, pathological ovarian samples should be assessed to dissect the mechanisms of ovarian lesions, even ovarian cancer, laying a foundation for ovarian therapy.

## Conclusion

In summary, this is the first systematic exploration of proteome and modified proteome in premenopausal and postmenopausal ovarian samples. With a comprehensive proteome and acetyl-proteome analysis, the changes of protein expression and acetylation in premenopausal and postmenopausal ovarian tissues were investigated. Then, the differential changes of molecular functions, biological processes, cellular components, pathways and motifs during menopausal transition were evaluated in detail. Specific changes in global protein or acetylated protein expression levels may constitute potential markers of ovarian pathology and help develop treatments for menopausal symptoms and ultimately advance women’s health care.

## Additional files

**Additional file 1: Table S1.** Global proteomic analysis of ovarian tissues from perimenopausal and postmenopausal women by LC–MS/MS using TMT quantification.

**Additional file 2: Table S2.** Differential protein expression in postmenopause and premenopause samples.

**Additional file 3: Table S3.** Acetylated proteome analysis of ovarian tissues from perimenopausal and postmenopausal women by LC–MS/MS using TMT quantification and antibody-affinity purification.

**Additional file 4: Table S4.** Differential acetyl-protein expression in postmenopause and premenopause samples.

**Additional file 5: Figure S1.**

**Additional file 6: Figure S2.**
