# Supplementary figures and images for: Differential analysis of quantitative proteome and acetyl-proteome profiling between premenopausal and postmenopausal ovarian tissues
Source: Clin Proteomics. 2018 Nov 16;15:36. doi: 10.1186/s12014-018-9214-0 (PMC6238338; doi:10.1186/s12014-018-9214-0)

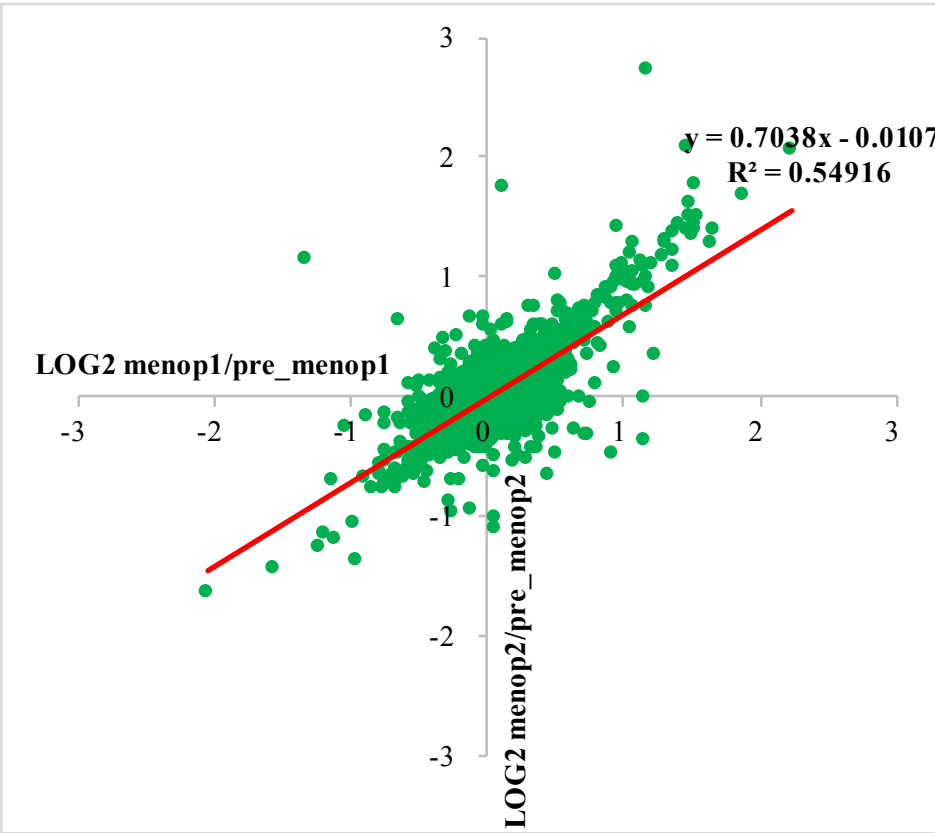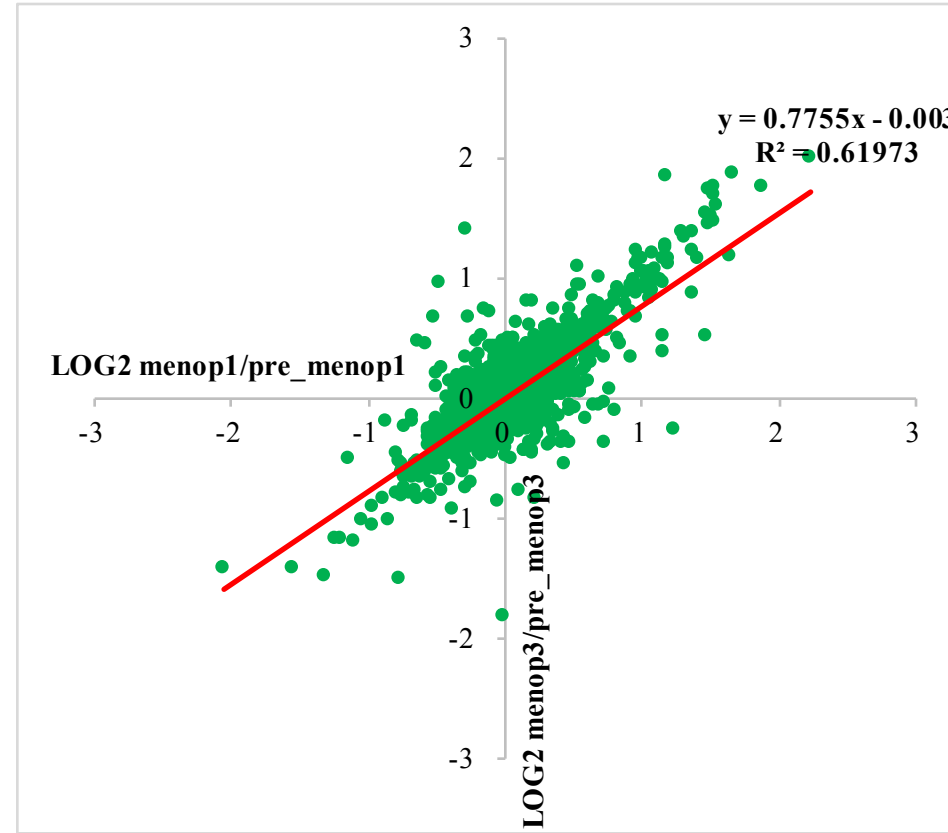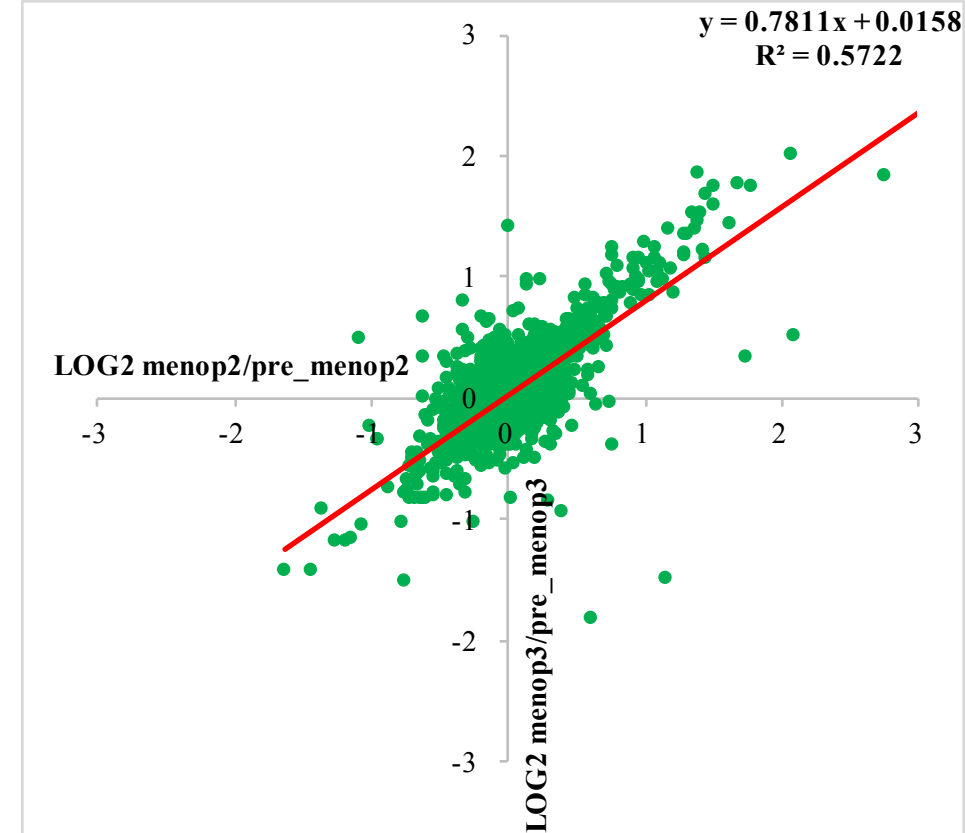

Supplement: Supplementary file 5 — Additional file 5: Figure S1. [file 12014_2018_9214_MOESM5_ESM.pdf]

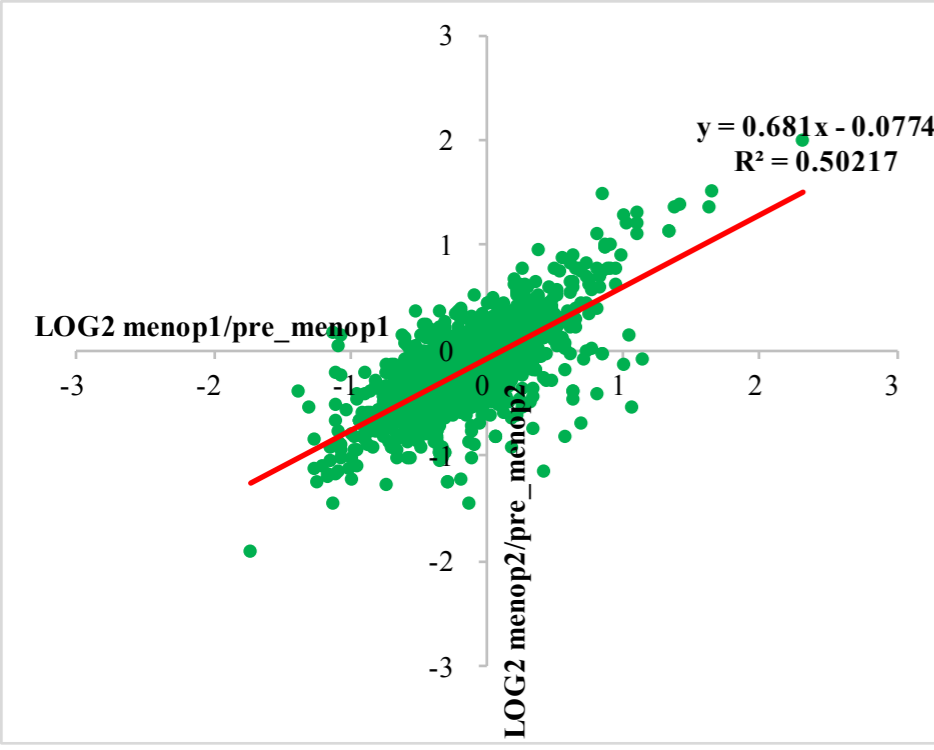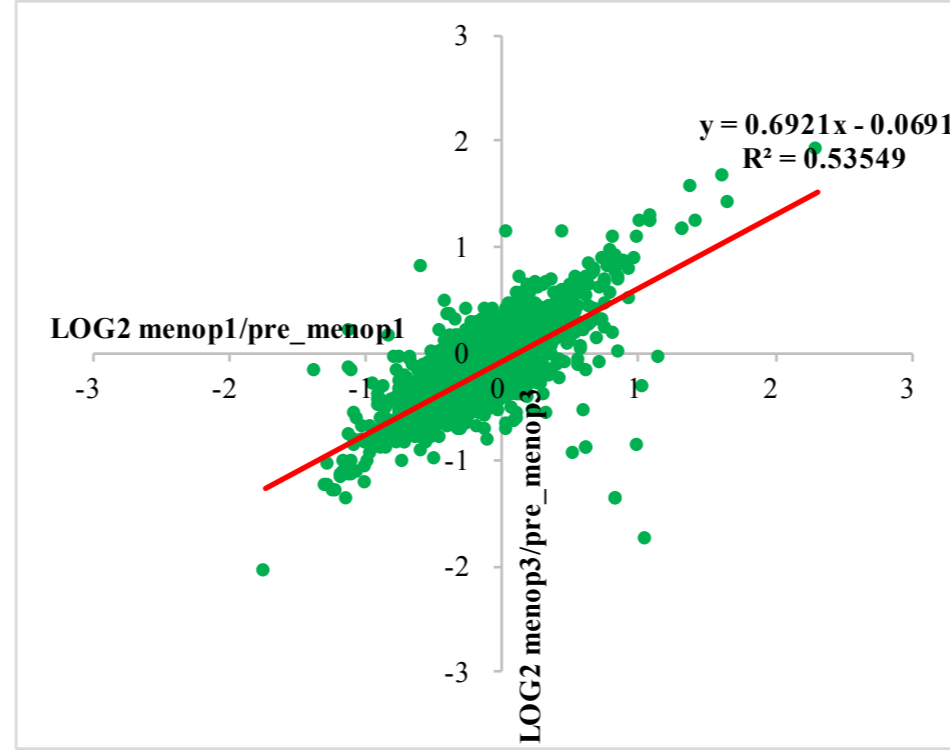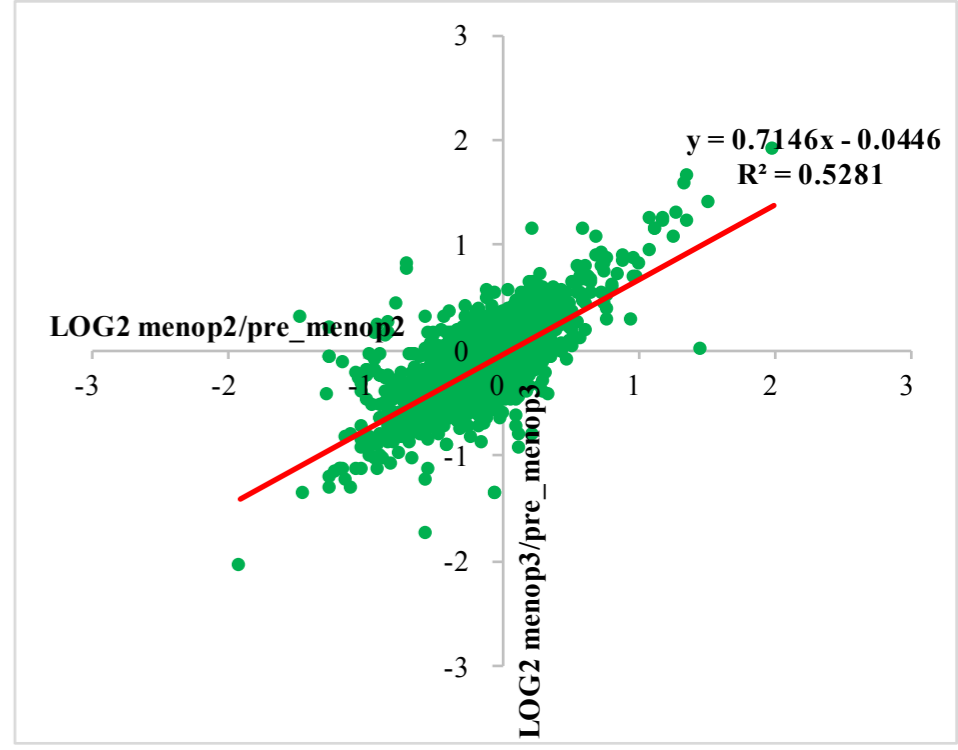

Supplement: Supplementary file 6 — Additional file 6: Figure S2. [file 12014_2018_9214_MOESM6_ESM.pdf]
